# Integration of model-based recursive partitioning with bias reduction estimation: a case study assessing the impact of Oliver’s four factors on the probability of winning a basketball game

**DOI:** 10.1007/s10182-022-00456-6

**Published:** 2022-07-04

**Authors:** Manlio Migliorati, Marica Manisera, Paola Zuccolotto

**Affiliations:** University of Brescia, Brescia, Italy

**Keywords:** Sport analytics, Basketball, Success drivers, MOB, Bias reduction GLM, Data mining

## Abstract

In this contribution, we investigate the importance of Oliver’s Four Factors, proposed in the literature to identify a basketball team’s strengths and weaknesses in terms of shooting, turnovers, rebounding and free throws, as success drivers of a basketball game. In order to investigate the role of each factor in the success of a team in a match, we applied the MOdel-Based recursive partitioning (MOB) algorithm to real data concerning 19,138 matches of 16 National Basketball Association (NBA) regular seasons (from 2004–2005 to 2019–2020). MOB, instead of fitting one global Generalized Linear Model (GLM) to all observations, partitions the observations according to selected partitioning variables and estimates several ad hoc local GLMs for subgroups of observations. The manuscript’s aim is twofold: (1) in order to deal with (quasi) separation problems leading to convergence problems in the numerical solution of Maximum Likelihood (ML) estimation in MOB, we propose a methodological extension of GLM-based recursive partitioning from standard ML estimation to bias-reduced (BR) estimation; and (2) we apply the BR-based GLM trees to basketball analytics. The results show models very easy to interpret that can provide useful support to coaching staff’s decisions.

**Supplementary Information:**

The online version contains supplementary material available at 10.1007/s10182-022-00456-6.

## Introduction

Some basketball experts argue that statistics are killing basketball. In our opinion, they are right only if the game is reduced to numbers, which cannot truly describe the game. Instead, an appropriate usage of those statistics as the input data for sound methods can transform numbers into useful information to support coaching staff’s decisions. In the last decades, publications on statistics in basketball have multiplied and tried to answer different research questions: forecasting the outcomes of a game, investigating how to prevent injuries, analysing players’ performance, identifying optimal game strategies (Zuccolotto and Manisera 2020). This manuscript focuses on the well-known Oliver’s Four Factors (Oliver 2004; Kubatko et al. 2007), which can be considered drivers of success in a basketball match, identifying team’s strengths and weaknesses in terms of shooting, turnovers, rebounding and free throws. The aim is to investigate how the weights of the Four Factors in determining the victory in a game change on the basis of the balance of power between the two teams competing in each match. With the goal of investigating the impact of each factor in estimating the probability of winning a game and, consequently, predicting the winner and simultaneously accounting for differences in class between the two teams, we applied the MOdel-Based recursive partitioning (MOB) algorithm (Zeileis et al. 2008) that, instead of fitting one global Generalized Linear Model (GLM), estimates several local GLMs considering subgroups of observations according to a learning algorithm based on recursive partitioning. To this purpose, we used real data from 19,138 matches of 16 National Basketball Association (NBA) regular seasons, from 2004–2005 to 2019–2020, and defined an index, used to partition observations with MOB, as the difference between victory relative frequencies in previously played games for the two teams competing in a match. The analysis highlighted (quasi) separation problems, introducing convergence problems in the numerical solution of the Maximum Likelihood (ML) estimation. To address this issue, we coupled MOB with Bias Reduction (BR) estimation (Firth 1993), which ensured finite estimates in the case of (quasi) separation situations (Kosmidis and Firth 2020).

This manuscript’s original contribution is:

1. From a methodological point of view, we propose an extension of GLM-based recursive partitioning from standard ML estimation to BR estimation by integrating MOB with BR GLM estimation, solving ML convergence problems and ensuring finite estimates when (quasi) separation occurs.
2. From an applied point of view, we introduce diversification of the Four Factors’ weights on the basis of the differences in class between the teams competing in a match, providing a valuable tool to coaching staff in analysing a match.

The manuscript is structured as follows: Sect. 2 offers a brief overview of the literature of statistics applied to basketball; Sect. 3 introduces the Four Factors, according to the Oliver’s definition, while Sect. 4 describes the dataset used in the application; Sect. 5 briefly summarises the MOB algorithm; Sect. 6 sums up the computational issues we encountered and proposes the original integrated solution mentioned in the above point 2; Sect. 7 reports the results from the application of the BR-based GLM trees to basketball data and proposes an analysis for both home and away matches, showing how the obtained results can be a valuable tool for coaching staffs. Section 8 concludes the manuscript. All implementations were made using the R programming language version 4.0.2 (R Core Team 2020) via R Studio version 1.2 (RStudio Team 2020), adopting the Tidyverse paradigm (Wickham 2019).

## Basketball and statistics

Today, data play a fundamental role in sport management, a world where a huge amount of money is invested but where fortuity plays a great role. This is the reason why a data-based approach was adopted early in each professional sport (Alamar 2013; Albert et al. 2017): to face different kinds of problems ranging from outcome prediction, injury prevention, performance measurement, player selection and much more. It was with the application of the data-driven approach described in Lewis (2003), centered on the Oakland Athletics baseball team, that analytics in sport actually entered the maturity phase. Then, quickly, data mining in sport was widely adopted and adapted in all professional sports, such as hockey, football, soccer and, of course, basketball (for example, Zuccolotto et al., 2017b, a; Groll et al., 2018; Metulini et al., 2018; Groll et al., 2019; Zuccolotto et al., 2019; Sandri et al., 2020). Basketball milestones of this analytics-based approach are pioneering works (Oliver 2004; Kubatko et al. 2007), where the famous Oliver’s “Four Factors to success” were introduced as four indexes containing a big amount of information. In this manuscript, we are interested in applying data mining to basketball as was shown in Zuccolotto and Manisera (2020), to understand how a match outcome (victory or defeat) is related to the Four Factors, and with which importance. The goal of this manuscript is not to produce predictions for next games to be played; instead, we aim to provide coaching staff with valuable tools to prepare a match, taking into account the strength of both teams. Considering the increasing volume of sport betting, it is clear why the number of attempts in accurately predicting games’ results is continuously growing, see for instance Bunker and Thabtah (2019) or Hubáček et al. (2019). Machine learning techniques for prediction have been widely applied (Haghighat et al. 2013), covering all professional sports, from horse races (Davoodi and Khanteymoori 2010) to hockey (Gu et al. 2016) and from American football (Purucker 1996; David et al. 2011) to soccer (Min et al. 2008; Tax and Joustra 2015; Carpita et al. 2019), just to name a few. In basketball, Loeffelholz et al. (2009) applied several kinds of Artificial Neural Networks (ANNs) to a dataset of 620 NBA games, to predict outcomes. A percentage of 74.33 of correct predictions, higher than experts’ percentage (claimed to be 68.67), was reported. Miljkovic et al. (2010) described how, among several machine learning algorithms, best results in both predicting the outcomes and calculating final match spread were produced by the Naïve Bayes approach. The Authors used 778 NBA games of the season 2009-2010, considering 141 features as input, and an accuracy of 67% is reported. In Cao (2012), data of five NBA seasons were analysed using ANN, Support Vector Machine (SVM), Naïve Bayes and logistic regression, with the latter producing the best prediction accuracy (about 70%) for the classification problem of predicting the winner of a game.

In a similar way, in Beckler et al. (2013) an accuracy of 73% was reported for NBA outcome prediction. The Authors applied linear regression, SVM, logistic regression and ANN on a dataset including seasons from 1991–1992 to 1996–1997. Cheng et al. (2016) applied the principle of maximum entropy in order to predict NBA playoff outcomes for seasons from 2007–08 to 2014–15, reporting an accuracy of 74.4%. Moreover, there are several betting websites suggesting NBA outcome predictions. As an example, the website Teamrankings (2020) proposes predictions about NBA match winners using four approaches and several sources (historical data, breaking news and trends). For the regular season 2019–2020, the prediction accuracy is claimed to be, on average 73%, and this percentage was obtained applying decision trees to games in November. The perspective of this manuscript is different: we are not interested in predicting outcomes; instead, we aim to understand how the Four Factors, used as predictors, can have a different importance in driving a match result on the basis of the strength balance between the two teams in each match. The philosophy is the same as in Migliorati (2020), where both decision trees and random forests were applied to a large NBA dataset to assess the importance of predictors constituted by both box scores’ statistics and the Four Factors. A similar philosophy can be found in Thabtah et al. (2019), where Naïve Bayes, ANN and logistic lodel trees were applied to a little dataset containing box score information about 430 observations of NBA final games from 1980 to 2017, and the number of defensive rebounds is identified as the most influential feature for victory.

## Four factors

The Four Factors are a set of derived statistics used for summarising the attitude of a team with respect to shooting, turnovers, rebounds and free throws. They are considered the key to winning a match, and were empirically weighted by Oliver (2004), as reported in Table 1.

**Table 1:** Empirical weight of Oliver’s Four Factors

Factor	Weight
Shooting	40%
Turnovers	25%
Rebounds	20%
Free throws	15%

The Four Factors are defined beginning from the concept of *possession*, i.e. the number of times a team gains control of the ball during a match, formulated in its simplest and most common way as follows (refer to Table 2 for variable meaning):

1 $$\begin{aligned} Poss = (P2A+P3A)+0.44*FTA-OREB+TOV \end{aligned}$$

In Eq. (1), FTA is weighted by 0.44, which is the fraction of free throws that end possessions, as estimated in the 2002–2003 through 2005–2006 seasons by Kubatko et al., 2007. Alternative and more complicated formulas have also been proposed in the specialised literature (Kubatko et al. 2007).

**Table 2:** Variables’ acronym meaning

Acronym	Meaning
P2A	2-Point field goals attempted
P3A	3-Point field goals attempted
FTA	Free throws attempted
P2M	2-Point field goals made
P3M	3-Point field goals made
FTM	Free throws made
OREB	Offensive rebounds
DREB	Defensive rebounds
TOV	Turnovers
POSS	Possessions

It is possible to define the Four Factors in the following analytic way:

1. Shooting, measured by effective Field Goals percentage:
  2 $$\begin{aligned} eFG\% = (P2M + 1.5*P3M) / (P2A + P3A) \end{aligned}$$
2. Turnover ratio, the number of turnovers (i.e. loss of ball) per possession:
  3 $$\begin{aligned} TO\_ratio = TOV / POSS \end{aligned}$$
3. Rebounds, defined by offensive rebounding percentage:
  4 $$\begin{aligned} OREB\% = OREB / (OREB + DREB) \end{aligned}$$
  where, in the denominator, the team offensive rebounds and the opponent team defensive rebounds are considered, respectively.
4. Free throws rate:
  5 $$\begin{aligned} FT\_rate=FTM / (P2A + P3A) \end{aligned}$$

For each match, the Four Factors for both the home team (marked with *ht* in the following) and the away team (*at*) can be computed, leading to eight factors that can be used.

## The dataset

The dataset analysed in this manuscript includes all the matches of the NBA regular seasons from 2004–2005 to 2019–2020 (until 11/03/2020, when the NBA was stopped for some months due to Covid-19).

We performed several analyses in order to find the best model for the manuscript’s aim. Let us consider the *g*-th match of a season composed of *G* matches. Among different indexes (season, conference, victory percentage, etc.), we have chosen to base the analysis on a statistic, named *diff*, computed for each match in each season and defined as the difference between the relative frequency of victories for *ht* and the relative frequency of victories for *at*:

6 $$\begin{aligned} diff_g=\frac{won\_matches_{ht,(g-k):(g-1)}}{played\_matches_{ht,(g-k):(g-1)}} -\frac{won\_matches_{at,(g-k):(g-1)}}{played\_matches_{at,(g-k):(g-1)}} \end{aligned}$$

where $won\_matches_{ht,(g-k):(g-1)}$ and $played\_matches_{ht, (g-k):(g-1)}$ indicate, respectively, the global number of matches won and played by the home team considering the last *k* matches - until the $(g-1)$-th match - played against all the teams met in the championship (both in home and away matches). Analogously, $won\_matches_{at, (g-k):(g-1)}$ and $played\_matches_{at, (g-k):(g-1)}$ indicate the global number of matches won and played by the away team in the last *k* matches. For some *g*’s, the two denominators in Eq. (6) may differ due to calendar differences in the championship schedule for the two considered teams *ht* and *at*.

The *diff* index ranges from $-1$ to 1. It equals 1 when the home team is absolutely the strongest between the two competing teams in that match, because the home team won all the games played so far in the season (against all the teams it encountered so far), while the away team never won. On the contrary, it equals $-1$ when the away team won all the games played so far and the home team never won.

In order to have a sufficient number of matches to compute the *diff* index, we modelled only the data from the second half of each season (so *g* starts from $G/2+1$ for every complete season) and computed (6), for each *g*, considering the number of matches won and played starting from the first match of the season ($g-k$ fixed and equal to 1 with *k* increasing as *g* increases, according to the increase in the amount of information available at the *g*-th match). The data from the second half of each season in the analysis include 9,569 observations. As Eq. (6) suggests, a possible alternative procedure to compute the *diff* index could be considering *k* adequately tuned in order to include only the past *k* matches (see, for example, Migliorati, 2021).

The *diff* index provides an easily interpretable way of summarising information about the results gained by the two competing teams in the last *k* matches of the season up to the match preceding the considered one, and can be interpreted as the difference in class between the two teams considered in each match.

The dataset was then split into a training set, including matches from seasons from 2004–2005 to 2017–2018 (8,469 observations, 88.5%), and a test set, including matches from seasons 2018–2019 and (part of) 2019–2020 (1,100 observations, 11.5%).

In addition, we applied two cross-validation procedures in order to check the stability of the obtained results, as will be explained in Sect. 7.3. Among more than 70 features available in the dataset, we pursued the manuscript’s aim by using (*i*) as the dependent variable, the *result* of the match (referred to the home team victory and identified with the two categories 1 and 0 for won and lost, respectively), (*ii*) as predictors, the Four Factors for both home and the away teams (calculated as defined in Sect. 3 using the R package BasketballAnalyzeR (Sandri et al. 2018; Sandri 2020) and the *diff* index, a variable directly addressing the strength difference of teams.

## MOdel based recursive partitioning (MOB)

MOB is an algorithm for recursive partitioning, as described in (Zeileis et al. 2008). In this approach, fitting is not made via a unique global model for the complete dataset, but instead in a local way, estimating model parameters on subsets of data defined by recursive partitioning. The MOB algorithm is composed of four steps:

1. A parametric model is fitted to all observations in a node using a set of regressor variables;
2. Parameter estimates are tested for overall instability with respect to a set of partitioning variables; if there are overall instabilities, the variable associated to the highest instability is selected;
3. The split value of the selected variable that locally optimises the objective function is chosen;
4. The node is split into child nodes, and the procedure is repeated for each child, until a stopping criterion is satisfied.

In this way a tree is built, where each leaf has associated a partition of the original observations. The subset of observations in each leaf is used for building a local fitting model, generally with a better fitting quality with respect to the global one.

Moreover, apart from predictive power, MOB provides the possibility to have tailored beta coefficients in regression, offering a higher interpretation value with respect to classical regression models.

MOB constitutes an interesting implementation of the Breiman’s “two cultures” paradigm (Breiman 2001), where the dependent variable *Y* is addressed by two sets of variables:

1. Variables $X_1, X_2, \dots , X_p$, constituting the set of predictors to be used for the stochastic data model, and
2. Variables $Z_1, Z_2, \dots , Z_l$, constituting the set of variables to be considered for recursive partitioning in building the tree, i.e. the algorithmic leg.

In our analysis, as already mentioned in the end of Sect. 4, the dependent variable *Y* is the *result* of a match, assuming value 1 if the home team won the match and 0 otherwise, and we will use the logistic regression model. The MOB algorithm is provided by the partykit R package (Hothorn and Zeileis 2015), and can be used with both predefined fitting function (e.g. linear regression or logistic regression) and integrating your own fitting function. Quality of fitting of models will be measured using the Receiver Operating Characteristic (ROC) curve with the Area Under the Curve (AUC) together with the accuracy index.

Starting from the concepts of sensitivity (the proportion of true positives correctly identified) and specificity (the proportion of true negatives correctly identified), a ROC plot displays the performance of a binary classifier, showing how both sensitivity and specificity change when the classification threshold ranges over all possible values. In other words, it shows the trade off in sensitivity and specificity for all possible thresholds.

In ROC context, AUC (Bradley 1997), ranging between 0 and 1, measures the performance of a classifier, where an higher AUC means a better classification.

The optimal threshold, identified according to (Youden 1950) as the cut-off that maximizes the distance from the ROC curve to the identity (diagonal) line, will be used to classify observations in the *result* categories 1 or 0 according to the estimated probabilities.

Like in GLMs, in the MOB algorithm an offset term can be added, in order to include an additional regressor in the linear predictor when the effect of this regressor is fixed or known a priori. Therefore, the coefficient of this additional term is not estimated but fixed. In our analysis, we will resort to the introduction of an offset term in order to investigate whether the impact of the Four Factors on the winning probability varies in matches according to the difference in strength of the two competing teams.

## A methodological extension of GLM-based recursive partitioning from standard maximum likelihood to bias reduction estimation

Logistic regression can produce a (quasi) separation problem (Albert and Anderson 1984) when a predictor or a linear combination of more predictors is associated with a single fixed outcome value from a certain value on. More precisely, if we consider the realisations $y_i$, with $i=1,\dots ,n$, of the *n* independent Bernoulli random variables and the *p*-dimensional covariate vector $x_i$ associated with each $y_i$, we have (Allison 2004, 2008):

- complete separation in the sample points $(y_1,x_1^T)^T,\dots ,(y_n,x_n^T)^T$ if there exists a vector $\gamma \in R^p$ such that $\gamma ^T x_i>0$ $\forall i$ with $y_i=1$ and $\gamma ^T x_i<0$ $\forall i$ with $y_i=0$;
- quasi-complete separation or (quasi) separation in the sample points if there exists a vector $\gamma \in R^p$ such that $\gamma ^T x_i\ge 0$ $\forall i$ with $y_i=1$ and $\gamma ^T x_i\le 0$ $\forall i$ with $y_i=0$;
- overlap if neither complete nor quasi-complete separation occurs.

Data separation is a necessary and sufficient condition for the maximum likelihood estimate to have at least one infinite-valued component (Albert and Anderson 1984). In (quasi) separation situations, standard maximum-likelihood estimation methods, such as the Iteratively Reweighted Least Squares (IRLS) (Green 1984) commonly used in logistic regression, can be numerically unstable and inferential results can be wrong when procedures are based on the estimates and the estimated standard errors (Mansournia et al. 2018).

A possible solution to obtain finite estimates in the presence of a (quasi) separation is to exploit alternative estimation methods aimed at reducing the estimation bias (Firth 1993). Such methods, implemented in the brglm2 package in R (Kosmidis 2020), work by maximising a penalised likelihood where the penalty is given by powers of the Jeffreys’ invariant prior and the estimation bias can be reduced by solving the mean bias-reducing adjusted score equations (Firth 1993; Kosmidis and Firth 2020), by solving the median bias-reduction adjusted score equations (Kenne Pagui et al. 2017) or by subtracting an estimate of the bias from the maximum likelihood estimates (Cordeiro and McCullagh 1991). Computationally, a quasi Fisher scoring algorithm is used. We incurred in warnings about the (quasi) separation when both classical logistic regression and MOB (using logistic regression as a fitting function) were used to fit data with *result* (won or lost) as the dependent variable, the Four Factors as predictors and some variables in the dataset as partitioning variables.

While for logistic regression the solution is ready and already implemented in the above mentioned brglm2 package in R, in MOB, only the standard maximum likelihood estimation procedure was available. Therefore, in this manuscript, we propose to integrate the MOB algorithm with a bias reduction method, namely the mean bias-reducing adjusted score approach (Firth 1993), ensuring finite estimates for some GLM (and among them Binomial response) (Kosmidis and Firth 2020), even in cases where the maximum likelihood estimates are divergent (as for separation problem).

We implemented this solution in R by integrating MOB with the R package brglm2 (Kosmidis 2020; Kosmidis et al. 2020) in order to obtain finite estimates in each of the logistic regression models estimated in the MOB algorithm. This adds another context to the list of the different application contexts where the reduced-bias estimator can favourably be used (see, for example, Kosmidis and Firth, 2020 for a list of diverse application areas). This integration is now natively availabe in the package partykit as introduced by its authors.

## Application of the MOB algorithm with brglmFit to the NBA dataset

In this section, we summarise the results obtained by applying the MOB algorithm with brglmFit (from package brglm2) to the NBA dataset.

We considered two models:

1. The first model aimed at helping the coaching staff when preparing the home match; the winning probability of the home team is modelled as function of the home team’s Four Factors.
2. The second model aimed at supporting the preparation of the away matches, based on the opponent’s Four Factors as predictors. In this second model, the winning probability of the away team is modelled as function of the away team’s Four Factors.

In both cases, the partitioning variable used in MOB is the *diff* index, which is the difference of victory relative frequencies between the home and the away team, as defined in Eq. (6).

The first results, described in Subsects. 7.1 and 7.2, suggested to investigate in much detail whether and how the impact of the Four Factors on the winning probability varies in matches according to the difference in strength of the two competing teams. In order to reveal any structural difference among the impacts of the Four Factors on the winning probability in the tree leaves, in Subsect. 7.3 we included the *diff* index as an offset term in the estimation of the GLM tree. In this way the parameter of the *diff* index is fixed globally for all observations while investigating, by estimating the tree, if and how the Four Factors’ parameters vary locally across subgroups of observations.

To quantify the global effect of the *diff* index we first estimated, on all observations, a logistic regression model with the winning probability as the dependent variable and the *diff* index as the sole regressor. The resulting estimated coefficient of the *diff* index measures the (a priori known) contribution of *diff* and, multiplied by the *diff* index itself, has then been used as the offset term in the GLM tree. The choice to model home and away matches separately is due to two main reasons: (1) we aimed to deliver two separate tools to the coach or team management as support when deciding the best game strategy, as explained above; and (2) including all the information for the home and away teams in one single dataset (and incorporating team information into the model) resulted in a model able to fit the data nearly perfectly (in particular, including the first factor of both teams makes it very easy to identify which team scored the most).

### A model for the home matches

In this subsection we analyse a model suitable for preparing home matches. The dependent variable *Y* is the match *result*, with possible categories 1 = won and 0 = lost, referred to the home team; the predictors are the home team’s Four Factors; partitioning is based on the *diff* index.

**Fig. 1:**
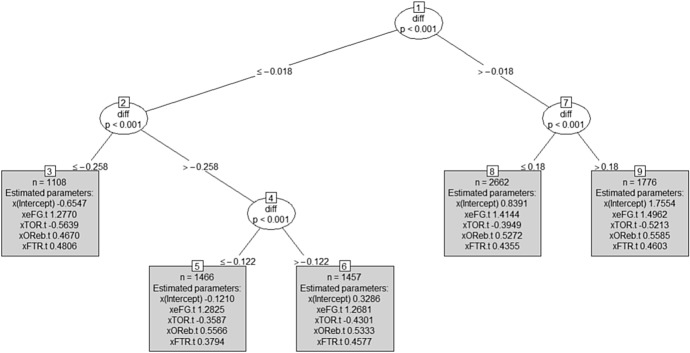
MOB tree for the home team model (training set): in the leaves, coefficient’s estimates are shown together with each subset size *n*

*Fitting the ‘home model’ to the training set* The model estimated on the training data is displayed in Fig. 1.

Each leaf has its own logistic regression model, which is fitted to the observations in that node.

**Fig. 2:**
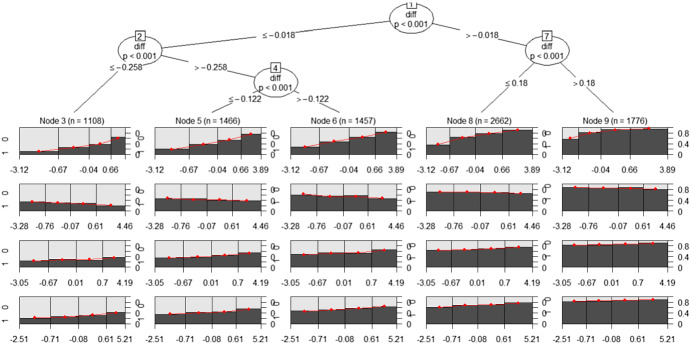
MOB tree for the home team model (training set): for each leaf, a set of four spinograms, one for each factor (from top to bottom: shooting, turnovers, rebounds and free throws, respectively), is depicted

Figure 2 shows the specific model associated to each leaf summarised via four spinograms, one for each factor (shooting, turnovers, rebounds and free throws, respectively), offering a simple tool to better interpret the results.

The spinogram is a plotting style provided by the partykit package for logistic regression, which shows how the success probability of the dependent variable $Pr(Y=1)$ (on the *y*-axis) varies on the basis of an independent variable on the *x* axis. The stacked bars’ widths correspond to the relative frequencies of the values of the independent variable (in classes), while the bars’ heights correspond to the conditional relative frequencies of $Y=1$, which are the success probability estimates, in every class of the independent variable (defined according to a quantile subdivision, here quartiles).

Looking at the first leaf on the left (Node 3) in Fig. 2, which includes observations with negative *diff* (the away team won more than the home team up to the current match, so we can suppose that the away team is stronger than the home team), we can observe that the model highlights the difficulty for the home team to find a winning strategy. Only a really high shooting percentage can give the home team a hope of victory. This suggests a really high weight of the shooting factor in the game strategy in this situation.

In the opposite extreme (first leaf on the right), Node 9 contains observations with positive *diff* ($>0.18$). For the games included in this node, we can suppose that the home team is stronger than the away team. Here, the model confirms that, as expected, winning probabilities are really high, regardless of the Four Factors. It is a good situation in which the coach can try new strategies and tactics, or let play second lines allowing first lines to stand.

Middle nodes include more balanced matches, with fighting teams having similar victory percentages in the matches played up to the current match. For example, looking at Nodes 5 and 6, the spinograms suggest that a good shooting factor is mandatory to win, otherwise, with high probability, the home team will be defeated; for the other three factors, winning probability tends to be substantially flat.

*Testing the ‘home model’* Testing is based on the test set, containing 1,100 matches (11.5%) from the second half of the regular seasons 2018-2019 and 2019-2020. In our training set the home teams win more often (59.81% of wins, that is $Y=1$) than the away teams; wins prevalence has been considered in prediction to balance the dataset.

**Fig. 3:**
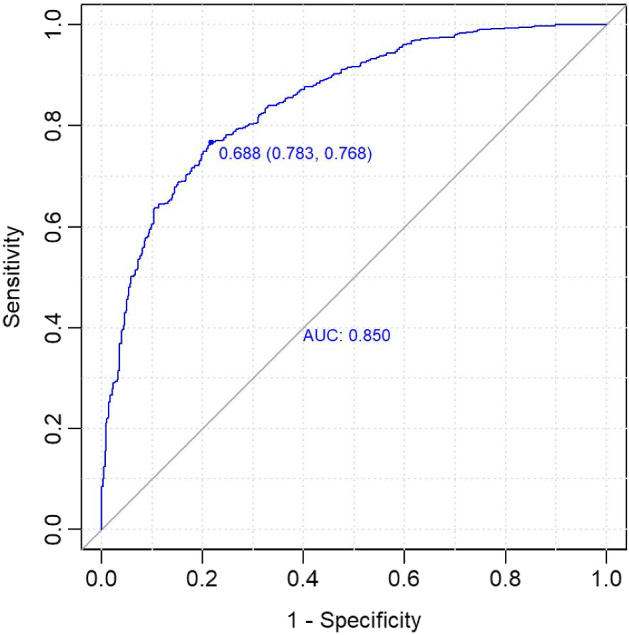
ROC/AUC analysis for the ‘home model’ (test set)

The results from the ROC/AUC analysis in the test set, performed using the pROC package (Robin et al. 2011), show a good fit, as displayed in Fig.  3: AUC equals 0.85, and for the selected model (with a classification threshold equal to 0.688), the accuracy is 0.7745. This high value of the accuracy index, as well as those described in the following, is justified because this model and the subsequent ones are not models for predicting the match result but for explaining the influence of the four factors on the match result; indeed, the covariates refer to the current game.

Size and accuracy for single leaves, i.e. for each locally fitted model, are reported in Table 3.

**Table 3:** Size and accuracy of local models for the “home model” (test set)

Node	Num	Accuracy
3	176	0.7955
5	169	0.7337
6	191	0.7382
8	334	0.7515
9	230	0.8522

As expected, accuracy is higher in extreme situations (when one of the two teams had much better performance than the other one up to the current match), and is lower (but still high) in balanced games, when the strength of the two teams is similar (*diff* is around 0).

### A model for the away matches

In this subsection we fit a model suitable in approaching the away matches. The focus is here on the away team so the dependent variable is the away team *result* and the predictors are the away team’s Four Factors; partitioning is still based on the *diff* index.

**Fig. 4:**
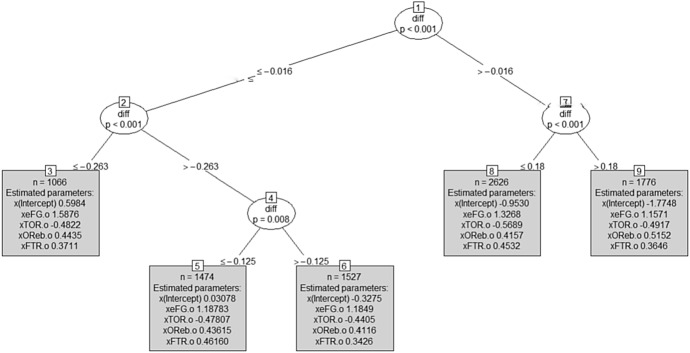
MOB tree for the away team model (training set): in the leaves, coefficient’s estimates are shown, together with each subset size n

**Fig. 5:**
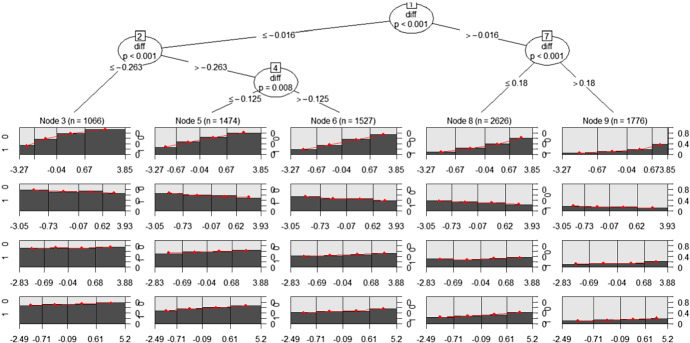
MOB tree for the away team model (training set): for each leaf, a set of four spinograms, one for each factor (from top to bottom: shooting, turnovers, rebounds, free throws, respectively), is depicted

*Fitting the ‘away model’ to the training set* The model estimated on the training set is displayed in Figs. 4 and 5.

Results from the home and away models are very similar, as expected, in terms of both partitioning and estimated models.

**Fig. 6:**
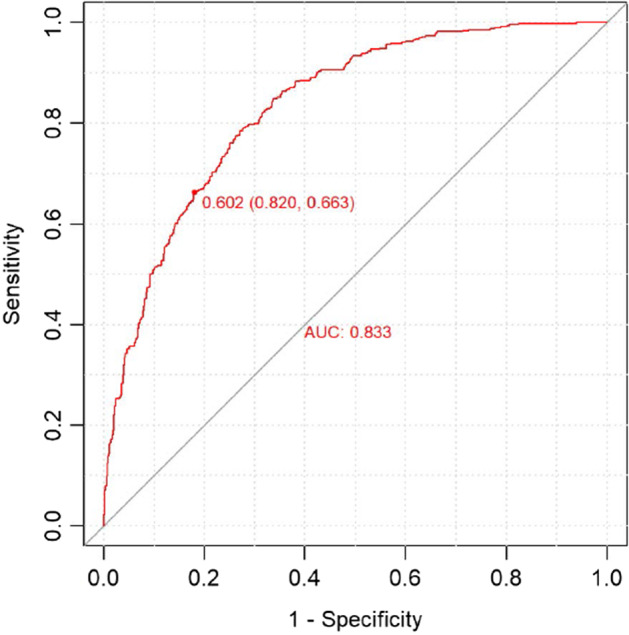
ROC/AUC analysis for the ‘away model’ (test set)

*Testing the ‘away model’* Results from the ROC/AUC analysis in the test set are displayed in Fig. 6, with an AUC of 0.833; for the selected model (with classification threshold equal to 0.602), accuracy is 0.7509.

Size and accuracy for single leaves, i.e. for each local fitting model, are reported in Table 4.

**Table 4:** Size and accuracy of local models (testing dataset), the away team

Node	Num	Accuracy
3	169	0.7515
5	172	0.7267
6	200	0.755
8	329	0.7173
9	230	0.813

### The GLM tree with an offset term

**Fig. 7:**
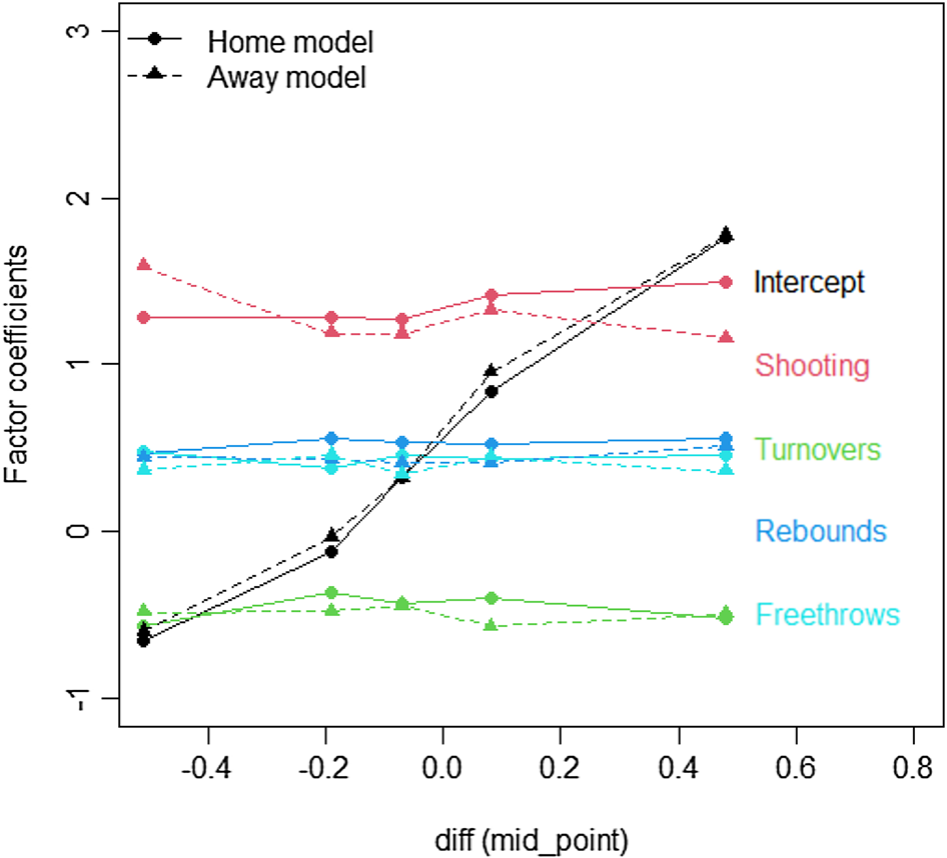
Estimated Four Factors’ coefficients (*y*-axis) as a function of the node mid-points of the partitioning variable (*diff* index, *x*-axis)

The results in Subsects. 7.1 and 7.2 suggest that the splits in both trees are mainly driven by differences in the intercept and that the remaining differences are mostly random variations. This is confirmed by Fig. 7, representing the estimated Four Factors’ coefficients as functions of the node mid-points of the partitioning variable (the *diff* index).

**Fig. 8:**
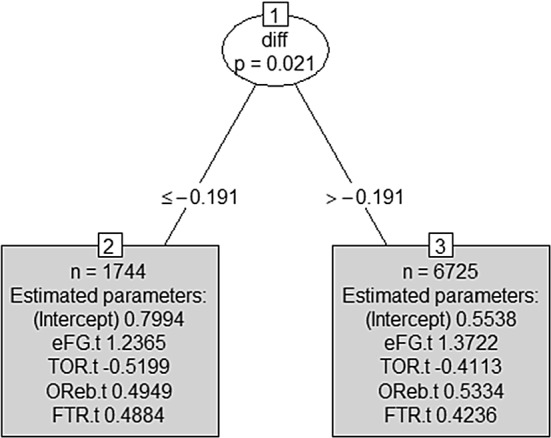
MOB tree for the home team model (training set) with the *diff* index as an offset term

**Fig. 9:**
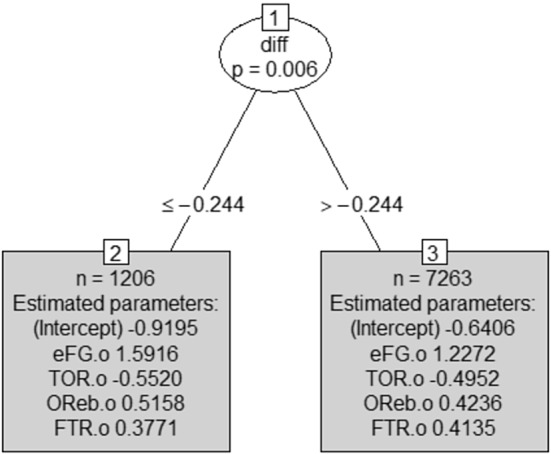
MOB tree for the away team model (training set) with the *diff* index as an offset term

Figure 7 suggests that the splits in both the home and away models are driven by differences in the intercept and not by differences in the Four Factors’ coefficients, which appear to be nearly constant across different nodes. This implies that the winning probability increases or decreases with the *diff* index, but the Four Factors do not seem to contribute much. The analysis of the impacts of the Four Factors on the winning probability in the tree leaves was repeated for both home and away GLM tree models; results are reported in Figs. 8 and 9, which suggest that in both situations, only one split is enough to distinguish structural differences in the impact of Four Factors on the winning probability.

**Fig. 10:**
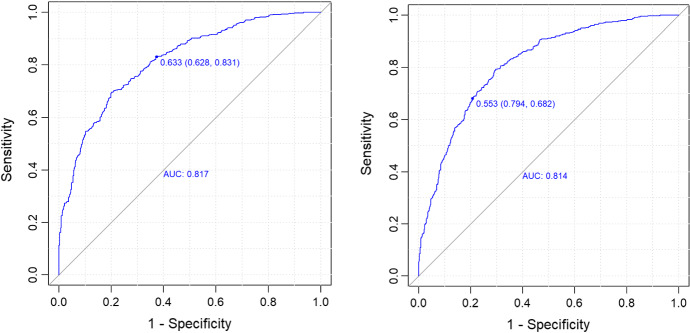
ROC/AUC analysis for the ‘home model’ (left) and the ‘away model’ (right), with the *diff* index as an offset term (test set)

Figure 10 shows the results from the ROC/AUC analysis in the test set for the home model (left) and the away model (right). AUC equals 0.817 and 0.814 in the home and in the away model, respectively. For the selected models (classification thresholds equal 0.633 and 0.553, respectively), accuracy is 0.7418 in the home model and 0.7445 in the away model.

To check stability of the obtained results we applied cross validation to both home and away team models, following two different approaches:

1. a classical 4-fold validation; for both the home and away team models, 3 out of 4 models produced one single split like in the models proposed in the manuscript, and one model produced no splits. The average values of some model results, reported in Tables 5 and 6 for the home and away model, respectively, show a substantial stability.
2. a “rolling” cross validation procedure. Inspired by Hyndman and Athanasopoulos, 2021, we defined an “ad hoc” cross validation procedure that is adapted from time series validation (our data are not classical time series, being not equidistant in time). In this approach, cross validation starts with a training dataset constituted by $n$ seasons and a test set composed of season $n+1$. Then, season $n+1$ is added to the training dataset, and the model trained on seasons from 1 to $n+1$ is tested on season $n+2$. The process is repeated until all seasons are considered. We started with $n$ equal to 10, in order to have a sufficiently large training set. Since our entire dataset was composed of 16 seasons, our “rolling” cross-validation procedure consisted of 6 runs. In all of them, for both home and away team models, one single split was produced and also in this case results, reported in Tables 5 and 6 for home and away model, respectively, confirm a substantial stability of the models’ results.

In detail, Tables 5 and 6 for the home and away model, respectively, report the average values for AUC, estimated coefficients of the two models expressed by left and right leaves, respectively, and the split threshold, considering the results from the 4-fold cross validation and the proposed “rolling” cross-validation. Results from the original models, also reported in Tables 5 and 6, are very close to the average values from both 4-fold cross validation (based on averaging the 3 models producing splits) and the “rolling” cross validation, confirming the stability of results.

**Table 5:** Results obtained from the original model for the home matches, and average results from the 4-cross validation (CV) and the proposed “rolling” cross validation (Rolling CV) procedures

Values	Original model %	CV	Rolling CV %
AUC	0.8170	0.8043	0.8083
intercept left leaf	0.7994	0.7490	0.8169
coefficient eFG.t left leaf	1.2365	1.2672	1.2414
coefficient TOR.t left leaf	−0.5199	−0.5264	−0.5428
coefficient OReb.t left leaf	0.4949	0.5230	0.4748
coefficient FTR.t left leaf	0.4884	0.4800	0.4836
intercept right leaf	0.5538	0.4772	0.5696
coefficient eFG.t right leaf	1.3722	1.3333	1.3743
coefficient TOR.t right leaf	−0.4113	−0.3983	−0.4086
coefficient OReb.t right leaf	0.5334	0.5443	0.5294
coefficient FTR.t right leaf	0.4236	0.4349	0.4264
split threshold	−0.1910	−0.1781	−0.1917

**Table 6:** Results obtained from the original model for the away matches, and average results from the 4-cross validation (CV) and the proposed “rolling” cross validation (Rolling CV) procedures

Values	Original model %	CV	Rolling CV %
AUC	0.8140	0.7945	0.8069
intercept left leaf	−0.9195	−1.0074	−0.9236
coefficient eFG.o left leaf	1.5916	1.4928	1.5391
coefficient TOR.o left leaf	−0.5520	−0.4626	−0.5309
coefficient OReb.o left leaf	0.5158	0.4488	0.4833
coefficient FTR.o left leaf	0.3771	0.3613	0.3835
intercept right leaf	−0,6406	−0.6566	−0.6316
coefficient eFG.o right leaf	1.2272	1.2401	1.2257
coefficient TOR.o right leaf	−0.4952	−0.4923	−0.4868
coefficient OReb.o right leaf	0.4236	0.4543	0.4172
coefficient FTR.o right leaf	0.4135	0.4284	0.4176
split threshold	−0.2440	−0.2327	−0.2360

The two trees displayed in Figs. 8 and 9 are very easy to read and their straightforward interpretability is an added value when these tools are delivered to coaches or the team management as support for their strategic decisions. The home model in Fig. 8 will drive decisions when a team is playing a home match, while the away model in Fig. 9 will support decisions if the team is playing away.

First of all, interestingly in both trees the threshold is not zero but is negative. This suggests that the threshold is favorable to the home team; indeed, in both trees, the leaf on the right contains matches where the home team is expected to have an advantage over the away team. On the contrary, in all the matches belonging to the left leaf, the home team is expected to have a disadvantage. The bonus for the home team, related to the negative threshold, is due to the presence, in the right leaf, of matches with a slightly negative *diff* deriving from a relative frequency of victories for the home team -up to that match- (slightly) lower than the relative frequency of victories for the away team.

Concerning the estimated coefficients, results show that, in general, the first factor offers a high contribution in winning the match, as expected, while the contributions of other three factors are lower and roughly equal to each other. This contradicts, in some way, the weights proposed by Oliver (Table 1).

Looking for differences in the estimated coefficients of the right and left models, we see that in matches where our team is expected to have an advantage (right leaf in the home model in Fig. 8 if we are playing at home and left leaf of the away model in Fig. 9 if we are playing away), the impact of the first and third factors on our winning probability is higher than in matches where our team is expected to suffer more. On the contrary, the impact of the fourth factor is lower. In other words, the best strategy for winning a game when we are the favorite team (according to the past history of games as measured by the *diff* index) is to focus on offensive abilities. On the other side, in matches where we are not the favorite team (left leaf in Fig. 8 and right leaf in Fig. 9), offensive skills are less crucial and a defensive strategy, blocking the opponent’s attack, and counting on free throws, can lead to win.

In future research, these simple models can be easily and profitably integrated with other partitioning variables, if available, for example, accounting for differences in teams’ positions in the conference (or division) rankings or in some offensive or defensive characteristics.

It is worth noting that the inclusion of an offset term in the GLM tree can be alternatively pursued by using the PALM tree algorithm for partially additive GLM trees (Seibold et al. 2019), with the difference that PALM integrates the estimation of the global and local effects in an iterative alternating algorithm that iterates between the estimation of the global parameters (for a given tree structure) and the estimation of the tree structure for a given global parameter vector.

## Conclusions

In this manuscript, we present a methodological innovation to solve the convergence problem of Maximum Likelihood estimation due to (quasi) separation issues we encountered in an empirical analysis of basketball data.

The proposed solution consists in the integration of MOB with Bias Reduction GLM estimation provided by the brglm2 package, ensuring finite estimates in (quasi) separation situations. We used this solution in the application of MOB to a large NBA dataset, in order to investigate how the Oliver’s Four Factors importance changes on the basis of the balance of power between two teams competing in each match. Of course, if the aim of the study had been only the investigation of a basketball analytics issue, other statistical or algorithmic models (for example, Generalized Additive Models) could also serve the purpose, perhaps more easily.

We analysed two different models with the result of the match as the dependent variable. The first model can be used by the coaching staff to prepare home matches (using the home team’s Four Factors as predictors), the second one to make decisions about the best strategy in away matches (using the opponent team’s Four Factors).

In both cases, we used the *diff* index as the partitioning variable; it is defined, for each match, as the difference between victory relative frequencies for the home and the away team, considering previous games of the same season.

The whole dataset was used to calculate the *diff* index, but only matches of the second half of each season were used as observations in the model; second half seasons from 2004–2005 to 2017–2018 were used for training, while second half seasons 2018–2019 and (part of) 2019–2020 were used for testing.

Results illustrate how local models provided by MOB offer a good interpretation power, showing how the Four Factors’ weights only slightly change when the difference in class between the two teams involved in a match is considered.

Further research will be devoted to include other partitioning variables in the models, in order to add information on the offensive or defensive abilities of the two teams, or external information, such as, for example, changes in the teams’ rosters, changes in the coaching staff, position in the conference or division rankings. Other definitions of the *diff* index could also be explored, varying the k parameter in order to account for a rolling estimate of the *diff* index including, for example, the previous four or five games.

## Supplementary Information

Below is the link to the electronic supplementary material.

Supplementary file 1 (xlsx 10 KB)

## Data Availability

Data used in this manuscript have been made available by the data provider BigDataBall (www.bigdataball.com).

## Code availability (software application or custom code)

The R code and an example dataset are available. Functions in the R package BasketballAnalyzeR are freely available on CRAN.
